# Life Course Malaria Exposure and SARS‐CoV‐2 Seroepidemiology in Ugandan Adolescents: A Longitudinal Study Nested in a Birth Cohort

**DOI:** 10.1111/tmi.70148

**Published:** 2026-04-23

**Authors:** Ludoviko Zirimenya, Doreen Nayebare, Claire Baine, Jacent Nassuuna, Milly Namutebi, Christopher Zziwa, Denis Nsubuga, Fred Kiwudhu, Florence Akello, Josephine Tumusiime, Gloria Oduru, Grace Kabami, Hellen Akurut, Rachel Nakyesige, Agnes Natukunda, Anne Wajja, Lawrence Lubyayi, Simon Kimuda, Gyaviira Nkurunungi, Jennifer Serwanga, Katie J. Doores, Emily L. Webb, Alison M. Elliott

**Affiliations:** ^1^ Immunomodulation and Vaccines Focus Area, Vaccine Research Theme Medical Research Council/Uganda Virus Research Institute and London School of Hygiene and Tropical Medicine (MRC/UVRI and LSHTM) Uganda Research Unit Entebbe Uganda; ^2^ Department of Clinical Research London School of Hygiene and Tropical Medicine London UK; ^3^ Department of Infectious Diseases King's College London London UK; ^4^ Department of Infection Biology London School of Hygiene and Tropical Medicine London UK; ^5^ Viral Pathogen Theme MRC/UVRI and LSHTM Uganda Research Unit Entebbe Uganda; ^6^ Department of Immunology Uganda Virus Research Institute Entebbe Uganda; ^7^ International Statistics and Epidemiology Group, Department of Infectious Disease Epidemiology London School of Hygiene and Tropical Medicine London UK

**Keywords:** adolescents, COVID‐19, life course exposures, longitudinal study, malaria, SARS‐CoV‐2, serology, seropositivity, seroprevalence

## Abstract

**Background:**

COVID‐19 has had major global health impacts, yet reported morbidity and mortality have been lower in Africa despite serological evidence of widespread infection. Malaria has been proposed as a potential modifier of susceptibility to and outcomes of SARS‐CoV‐2 infection. We investigated whether prospectively measured life‐course malaria exposure was associated with serologically defined SARS‐CoV‐2 infection among adolescents in a malaria‐endemic setting and explored symptomatic COVID‐19 during follow‐up.

**Methods:**

We conducted a longitudinal study nested within the Entebbe Mother and Baby Study birth cohort in Uganda. Adolescents were enrolled and followed up 6 and 12 months later. Blood samples were collected at each visit for SARS‐CoV‐2 serology. Malaria exposure was defined using prospectively collected data on clinical malaria (from birth to 10 years), asymptomatic parasitaemia at annual visits and antibody responses to *Plasmodium falciparum* merozoite surface protein 2 (PfMSP‐2) and apical membrane antigen 1 (PfAMA‐1). The primary outcome was SARS‐CoV‐2 seropositivity, based on spike and nucleocapsid IgG responses, with seropositivity cut‐offs derived from pre‐pandemic samples. Secondary outcomes, including antibody concentrations and symptomatic PCR‐confirmed disease, were explored. Vaccinated participants were excluded from subsequent serological analyses. Logistic and linear regression models assessed associations with seropositivity and log_10_‐transformed antibody concentrations, adjusting for key demographic, socioeconomic and exposure‐related confounders.

**Results:**

Between August and November 2021, 476 adolescents (mean age 16.4 years (SD = 0.75); 205 (43.1%) female) were enrolled. Overall, 12.0% had ever had asymptomatic parasitaemia, and 61.3% had experienced clinical malaria. SARS‐CoV‐2 seropositivity among unvaccinated participants increased from 63% at baseline to 89.3% at 6 months and 94.9% at 12 months; no PCR‐positive cases were reported. Multivariable analyses showed no consistent association between malaria exposure and SARS‐CoV‐2 seropositivity or antibody concentration levels.

**Conclusions:**

We found no consistent evidence that life‐course malaria exposure was associated with SARS‐CoV‐2 infection as measured by serology or antibody levels.

## 1 Background

The emergence and global spread of COVID‐19 resulted in profound public health consequences. For example, between 2019 and 2021, global life expectancy declined by an estimated 1.6 years [1]. Although the acute phase of the pandemic has passed, COVID‐19 remains a persistent threat with evolving variants. In response, the World Health Organization (WHO) recommends that countries maintain and strengthen COVID‐19 surveillance and response infrastructure to mitigate future outbreaks [2].

In Africa, the first case of COVID‐19 was reported in Egypt on 14 February 2020 [3]. As of 7 December 2025, the WHO reported an estimated 779,005,823 cumulative confirmed cases of COVID‐19 and 7,107,181 associated deaths globally, with Africa accounting for approximately 1.2% of reported cases and 2.5% of deaths [4]. Notably, serological studies suggest that the true burden of SARS‐CoV‐2 infection in Africa is substantially higher than case counts indicate. A systematic review and meta‐analysis of 151 seroprevalence studies conducted between January 2020 and December 2021 estimated that 65.1% of Africans had SARS‐CoV‐2 antibodies (95% CI: 56.3%–73.0%), comparable to levels reported in other global regions that had significantly higher numbers of reported cases [5].

The discrepancy between reported cases and seroprevalence has prompted several hypotheses. Limited testing capacity has often been cited as a possible explanation for the low number of reported COVID‐19 cases in Africa [3]. However, findings from a randomly sampled population‐based household serosurvey conducted in Ethiopia between July and September 2020 challenged this assumption, reporting seroprevalence rates of less than 5% in urban areas and under 2% in rural areas. These findings suggest that slower community spread, rather than under‐detection alone, may have contributed to the lower reported case counts, particularly early in the pandemic [6].

Another proposed explanation is a high prevalence of asymptomatic infections in Africa. A systematic review and meta‐analysis estimated that 64.3% (95% CI: 56.7%–71.6%) of infections in Africa were asymptomatic, the highest proportion of any region globally, compared with 40.0% (95% CI: 27.4%–53.3%) in the Americas, 28.1% (95% CI: 19.0%–38.1%) in Europe and 18.1% (95% CI: 13.2%–23.5%) in Asia [7].

Chronic exposure to endemic infections such as malaria has also been proposed as a potential biological contributor to the low burden of COVID‐19 in Africa. Malaria remains highly endemic in sub‐Saharan Africa (SSA), which accounted for 94% of the estimated 263 million malaria cases reported globally in 2023 [8]. Mechanistic studies provide biological plausibility, demonstrating that malaria modulates immune function. *P. falciparum* impairs dendritic cell‐mediated priming of adaptive immunity, leading to reduced CD+ T‐cell expansion, altered T‐B cell interactions, and blunted antibody responses to unrelated antigens [9]. Chronic exposure is further associated with reduced IFN‐γ‐producing T cells, immune exhaustion and impaired vaccine‐induced immunity [10]. In parallel, malaria disrupts B‐cell memory by driving expansion of atypical memory B cells with reduced proliferative and antibody‐producing capacity, potentially compromising responses to heterologous infections [11, 12].

Malaria exposure has therefore been hypothesised to influence immune responses to SARS‐CoV‐2, potentially contributing to a higher prevalence of asymptomatic or mild infections in malaria‐endemic populations [13, 14]. Evidence to date, however, has been mixed, with some studies suggesting a protective effect of malaria [15] and others reporting no association [16]. We therefore set out to investigate the association of life‐course malaria with serologically defined SARS‐CoV‐2 infection, as well as with COVID‐19 disease presentation. Understanding these potential interactions is important in malaria‐endemic regions where overlapping exposures may modulate both infection risk and disease outcomes.

## 2 Methods

### 2.1 Study Design

This longitudinal study was nested within the Entebbe Mother and Baby Study (EMaBS) birth cohort, whose study area comprises Entebbe Municipality and Katabi subcounty, a peninsula of Lake Victoria, Uganda. Malaria transmission in this urban setting is heterogeneous, resulting in substantial variation in cumulative malaria exposure among participants [17]. Established in 2003 as a randomised trial (ISRCTN32849447), EMaBS was originally designed to assess whether treating helminth infections during pregnancy and early childhood influences immune responses to vaccines, the incidence of infectious diseases and allergy‐related outcomes [18]. To address these aims, extensive data on malaria were prospectively collected from study participants.

### 2.2 Study Setting and Participants

EMaBS participants still under active follow‐up, aged 15–18 years, were invited to take part in this nested study, Impact of *co*‐infections and *ho*st genetics on susceptibility to SARS‐CoV‐2 infection and COVID‐19 disease severity in well‐characterised *st*udy cohorts in Uganda (CoHost). They were enrolled in the study if written informed consent was provided by the parent or guardian, and written informed assent was provided by the child (or informed consent for emancipated minors). Informed consent was obtained directly from participants aged 18 years.

Between 2020 and 2022, Uganda experienced three distinct COVID‐19 pandemic waves of the COVID‐19 pandemic: Wave 1 (Alpha virus variant) from December 2020 to January 2021; Wave 2 (Delta virus variant) from May 2021 to July 2021; and Wave 3 (Omicron virus variant) from December 2021 to February 2022 [19]. As the study was conducted between August 2021 and November 2022, it enabled assessment of how early‐life malaria exposure was associated with cumulative SARS‐CoV‐2 infection, as measured by longitudinal serology. Prospective follow‐up occurred largely during the period when the Omicron variant was circulating.

### 2.3 Data and Sample Collection

The EMaBS is a birth cohort that has prospectively collected detailed data on immunisations, infections, illnesses, immunological profiles and host genetics from pre‐birth, childhood and early adolescence up to and including the time of enrolment into this study.

**FIGURE 1:**
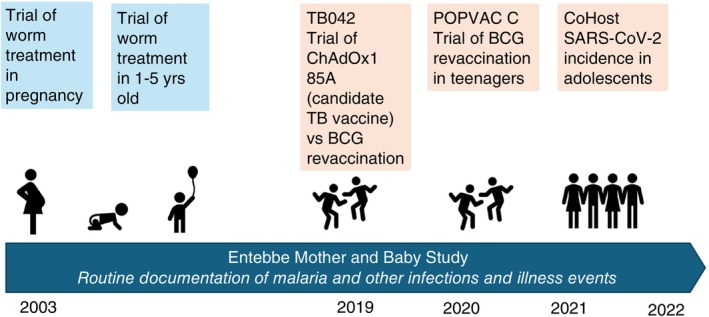
Showing the various trial interventions carried out prior to the CoHost study.

As part of EMaBS, several randomised interventions have been implemented (Figure 1), including trial treatments during pregnancy (praziquantel vs. placebo and albendazole vs. placebo), and quarterly albendazole treatment versus placebo when the children were aged 15 months to 5 years. In addition, subsets of study participants were enrolled in two further randomised controlled trials. In the Tuberculosis Vaccine 042 (TB042) trial (NCT03681860), 60 teenagers were randomised 1:1 to receive ChAdOx1 85A followed by MVA85A (a candidate booster TB vaccine regimen) or BCG revaccination [20]. In the POPVAC C trial (ISRCTN10482904), 300 teenagers were randomised to receive BCG revaccination or no revaccination, followed by a series of vaccinations that included Yellow Fever, oral typhoid, tetanus/diphtheria and human papillomavirus vaccines [21]. Pre‐vaccination immune profile and post‐vaccination responses were measured as part of these trials.

The four main exposures for this study were:

1. *P. falciparum* clinical malaria ever versus never (from birth to 10 years)
2. Number of episodes of *P. falciparum* clinical malaria (from birth to 10 years)
3. Asymptomatic malaria parasitaemia (ever vs. never) based on annual visits at ages 1–5, and at 9 and 10 years.
4. Malaria serology (PfMSP‐2 and PfAMA‐1) measured 1 year prior to enrolment in CoHost.

Clinical malaria was defined as the presence of fever (axillary temperature ≥ 37.5°C) with concurrent parasitaemia confirmed by thick film microscopy, diagnosed during unscheduled visits when children presented unwell. Clinical malaria was investigated as an exposure in two ways, namely ever/never and the number of episodes. Asymptomatic malaria was defined as the presence of parasitaemia on a blood slide during scheduled annual visits in participants who were afebrile and clinically well at the time of sampling; this exposure was categorised as ever versus never for analysis. These captured documented infection during follow‐up. In addition, *Plasmodium falciparum* merozoite surface protein 2 (PfMSP‐2) and apical membrane antigen 1 (PfAMA‐1) specific antibodies were measured as serological markers of cumulative malaria exposure. Antibodies were quantified using an in‐house ELISA as previously described [22]. These serological data were available only for the subset of participants enrolled in the POPVAC C trial [21].

Potential confounders of the relationship between life course malaria exposure and SARS‐CoV‐2 were also assessed. Variables collected at the CoHost baseline included age, sex, ethnicity, socio‐economic status (generated using principal components analysis of household construction materials, size and items owned) [23], maternal occupation, current residence, participant education level, BCG scar status and self‐reported history of COVID‐19 symptoms and vaccination status. Prospectively collected variables during EMaBS follow‐up from birth to adolescence included strain of BCG vaccination administered at birth, helminth infection detected during childhood follow‐up to 9 years, clinically diagnosed allergic conditions during infancy and childhood up to 10 years (including asthma, eczema and allergic rhinitis, as defined in previous EMaBS publication [24]), lower respiratory tract infection from birth to 10 years, Kaposi's sarcoma‐associated Herpesvirus infection at 5 years, and age of primary infection with cytomegalovirus (CMV), norovirus and herpes simplex virus (HSV).

During the study, participants were asked to complete a simple monthly symptom/contact diary and to alert the study team if they experienced COVID‐19 symptoms or if they had recently been in contact with someone with COVID‐19 or with COVID‐19‐like symptoms. Additionally, study staff contacted participants by telephone every 2 weeks to assess recent COVID‐19‐compatible symptoms and potential exposure to a suspected or confirmed COVID‐19 case. Participants who reported COVID‐19 symptoms or possible exposure were advised to attend Entebbe Regional Referral Hospital for SARS‐CoV‐2 PCR testing. When the Ministry of Health introduced COVID‐19 vaccination for school‐aged children in August 2022 [25], the study protocol was amended to integrate this opportunity for immunological monitoring. The amendment enabled the study to document vaccination status. COVID‐19 vaccinations were administered at Entebbe Regional Referral Hospital.

The primary outcome was SARS‐CoV‐2 serostatus at each timepoint. Seropositivity was defined by IgG responses to spike and nucleocapsid proteins measured using an in‐house enzyme‐linked immunosorbent assay (ELISA), with seropositivity cut‐offs derived from pre‐pandemic samples. Secondary outcomes included antibody concentrations and an exploratory outcome was PCR‐confirmed COVID‐19 disease. Outcome data were collected longitudinally at three timepoints: baseline (Month 0), Month 6 and Month 12. At each visit, 5 mL of blood was collected for SARS‐CoV‐2 serology (described in Appendix S1).

### 2.4 Definition of Serostatus

S‐antibodies are generated following both natural infection and vaccination, since most COVID‐19 vaccines target the spike protein. N‐antibodies, in contrast, are generated only after natural infection and are known to wane more rapidly over time [26, 27].

The cut‐off for seropositivity (IgG) to both SARS‐CoV‐2 S‐ and N‐proteins was calculated using pre‐pandemic samples, which were run on the same plates as the study samples. Study participants were defined as seropositive if their antibody concentration exceeded the mean antibody concentration plus three standard deviations (mean + 3SD) from the pre‐pandemic samples. This is consistent with established practice for internally validated immunoassays [28].

Based on these definitions, serological status at each time point was classified as follows:

- Negative (S‐protein seronegative and N‐protein seronegative): no serological evidence of prior infection or vaccination
- Positive (S‐protein seropositive and N‐protein seropositive) or (S‐protein seronegative and N‐protein seropositive) or (S‐protein seropositive and N‐protein seronegative): evidence of previous SARS‐CoV‐2 infection

Participants who received COVID‐19 vaccination before enrolment or during follow‐up were excluded from serological analyses done on samples collected subsequent to the date of vaccination.

### 2.5 Originally Determined Sample Size Calculation

The study sample size was determined based on the power to identify risk factors for SARS‐CoV‐2 infection. The calculation was based on the WHO's predictive model for widespread community transmission of SARS‐CoV‐2 infection in the African region, which estimated an incidence rate of 22% per annum [29]. Based on this incidence rate, it was anticipated that approximately 80 participants would test positive during the 12‐month follow‐up period. To achieve 80% power to detect an odds ratio of ≥ 2.0 for common exposures measured in all participants, it was necessary to enrol 500 participants in the study.

### 2.6 Statistical Analysis

Data were entered into a REDCap database and analysed using Stata 19.5 (College Station, TX, USA). Baseline characteristics were summarised using proportions for categorical variables and means and standard deviations (SDs) for continuous variables. Seropositivity with 95% confidence intervals (CIs) was estimated for each time point (Month 0, Month 6 and Month 12) with participants who reported COVID‐19 vaccination at study enrolment excluded. At Month 12, participants who received the COVID‐19 vaccination (which occurred between Months 6 and 12) were also excluded. Distributions of SARS‐CoV‐2 S‐ and N‐ antibody levels were described as continuous variables. Crude associations between participant characteristics and SARS‐CoV‐2 seropositivity were examined using chi‐squared tests for categorical variables and unpaired two‐tailed *t*‐tests for continuous variables.

For the binary outcome of seropositivity, logistic regression was used to estimate associations with life‐course malaria exposures and other covariates. For the continuous outcomes (S‐ and N‐antibody concentrations), linear regression was used to assess associations with malaria exposures and other covariates. Because antibody concentrations were skewed, log_10_ transformation was applied prior to regression modelling.

Potential confounders included all variables described above. In addition, we examined the effects of previous randomised interventions from the original EMaBS trial on the outcomes. Multivariable logistic and linear regression models were then developed for each malaria exposure, adjusting for confounders that showed an association with the outcome at *p* < 0.1 in crude analysis for each time point, with participants reporting vaccination at baseline excluded from all analyses, and participants who were vaccinated between Months 6 and 12 excluded from Month 12 analyses. Variables other than those of malaria exposure were considered as potential confounders or for descriptive purposes, and analyses involving these variables were conducted to inform which should be included as confounders in multivariable models. Sensitivity analyses restricted to anti‐nucleocapsid seropositivity were performed to minimise potential misclassification due to vaccination or antibody waning.

## 3 Results

### 3.1 Study Population Description

A total of 499 adolescents were enrolled in the CoHost study between 30 August and 22 November 2021; of these, 476 (95.4%) participants had baseline serology samples and were previously unvaccinated against COVID‐19. Baseline characteristics are summarised in Table 1. The mean age of participants was 16.4 years (SD = 0.75), and 205 (43.1%) were female. 57 (12.0%) participants had ever tested positive for asymptomatic malaria at an annual visit, and 292 (61.3%) had ever had an episode of clinical malaria from birth to 10 years. Regarding clinical malaria episodes, 184 (38.7%) participants had no recorded episodes of clinical malaria from birth to 10 years, 97 (20.4%) experienced one episode, 121 (25.4%) had two to four episodes, and 74 (15.6%) had more than four episodes. Only 46 (9.7%) participants had ever undergone SARS‐CoV‐2 diagnostic testing, with one confirmed infection reported before study enrolment. In addition, 31 (6.5%) participants reported prior contact with a suspected or confirmed COVID‐19 case.

**TABLE 1:** Characteristics of the study population.

Characteristic	Categories	Participant no. (%) *N* = 476
Demographics		
Sex	Male	271 (56.9%)
	Female	205 (43.1%)
Age	15 years	62 (13.0%)
	16 years	189 (39.7%)
	17 years	210 (44.1%)
	18 years	15 (3.2%)
BCG strain at birth	Denmark	37 (7.8%)
	Russia	255 (53.6%)
	Bulgaria	151 (31.7%)
	Unknown	33 (6.9%)
BCG Scar at enrolment	Absent	206 (43.3%)
	Present	268 (56.3%)
	Missing	2 (0.4%)
BCG revaccination status in the POPVAC C Clinical trial at age 13–17 years [21]	No	139 (29.2%)
	Yes	138 (29.0%)
	Did not take part in POPVAC C	199 (41.8%)
TB042 trial randomisation at age 12–17 years	BCG revaccination	26 (5.5%)
	ChAdOx1 85A followed by MVA85A	27 (5.7%)
	Did not take part in TB042	423 (88.9%)
Original EMaBS trial intervention 1 for the mothers during pregnancy	Albendazole	239 (50.2%)
	Placebo	237 (49.8%)
Original EMaBS trial intervention 2 for the mothers during pregnancy	Praziquantel	229 (48.1%)
	Placebo	247 (51.9%)
EMaBS trial interventions for the child from 15 months to 5 years	Albendazole	246 (51.7%)
	Placebo	227 (47.7%)
	Not randomised	3 (0.6%)
Body Mass Index at enrolment	Underweight	62 (13.0%)
	Normal weight	370 (77.7%)
	Overweight or Obese	44 (9.2%)
History of clinically diagnosed allergy during infancy or childhood	No	365 (76.7%)
	Yes	111 (23.3%)
Malaria exposures *n* (%)		
Any asymptomatic malaria infection at ages 1–5 and 9 years	No	419 (88.0%)
	Yes	57 (12.0%)
Any clinical malaria episode from birth to 10 years	No	184 (38.7%)
	Yes	292 (61.3%)
Number of clinical malaria episodes from birth to 10 years	None	184 (38.7%)
	1 episode	97 (20.4%)
	2–4 episodes	121 (25.4%)
	> 4 episodes	74 (15.6%)
Log_10_ transformed PfMSP‐2 antibody level (Mean, SD), arbitrary ELISA units	2.30 (0.28)	
Log_10_ transformed PfAMA‐1 antibody level (Mean, SD), arbitrary ELISA units	1.48 (0.74)	
Other infections *n* (%)		
Any helminth infection at ages 1–5 and 9 years	No	352 (74.0%)
	Yes	124 (26.1%)
Kaposi's sarcoma‐associated Herpesvirus infection at 5 years	No	247 (51.9%)
	Yes	188 (39.5%)
	Missing	41 (8.6%)
History of Lower Respiratory Tract Infection from birth to 10 years	No	306 (64.3%)
	Yes	170 (35.7%)
Age of CMV infection	2 years and below	213 (44.8%)
	Above 2 years	58 (12.2%)
	Missing	204 (43.1%)
Age of HSV infection	2 years and below	111 (23.3%)
	Above 3 years	128 (26.9%)
	Missing	237 (49.8%)
Age of norovirus infection	2 years and below	156 (32.8%)
	Above 2 years	30 (6.3%)
	Missing	290 (60.9%)
History of COVID‐19‐like self‐reported symptoms prior to enrolment	No	463 (97.3%)
	Yes	13 (2.7%)
Social factors *n* (%)		
Contact with a suspected or confirmed COVID‐19 case prior to enrolment	No	445 (93.5%)
	Yes	31 (6.5%)
Highest level of participant's education	Primary	85 (17.9%)
	Secondary	391 (82.1%)
Maternal occupation COVID‐19 exposure risk at enrolment^a^	High risk	244 (51.3%)
	Moderate risk	76 (16.0%)
	Low risk	36 (7.6%)
	Unemployed	43 (9.0%)
	Other	77 (16.2%)
Current residence	Urban	396 (83.2%)
	Rural	79 (16.6%)
	Missing	1 (0.2%)
Social economic status (SES) at enrolment^b^	Lowest (poorest)	118 (24.8%)
	Low (poor)	133 (27.9%)
	Middle (moderate)	105 (22.1%)
	High (wealthiest)	112 (23.5%)
	Missing	8 (1.7%)

Abbreviations: BCG, Bacillus Calmette‐Guérin; CMV, cytomegalovirus; ELISA, enzyme‐linked immunosorbent assay; HSV, herpes simplex virus.

^a^ Maternal occupations were grouped by potential exposure risk into high risk (e.g., healthcare workers and public‐facing service jobs such as shopkeepers, salon attendants, market vendors), moderate risk (e.g., casual labourers, tailors), low risk (e.g., office‐based jobs), unemployed, and others (occupations not classifiable into the above categories).

^b^ Household SES was calculated using principal components analysis of household materials and assets.

Between Months 6 and 12, 152 (37.4%) participants unvaccinated at baseline and with serology available at Month 12 received at least one dose of the COVID‐19 vaccine through the MOH programme and were excluded from analyses at Month 12.

### 3.2 SARS‐CoV‐2 Seropositivity and Crude Associations

Based on the mean + 3SD approach, the cut‐off values for defining seropositivity were 106.04 arbitrary ELISA units for S‐protein IgG and 55.26 arbitrary ELISA units for N‐protein IgG.

**FIGURE 2:**
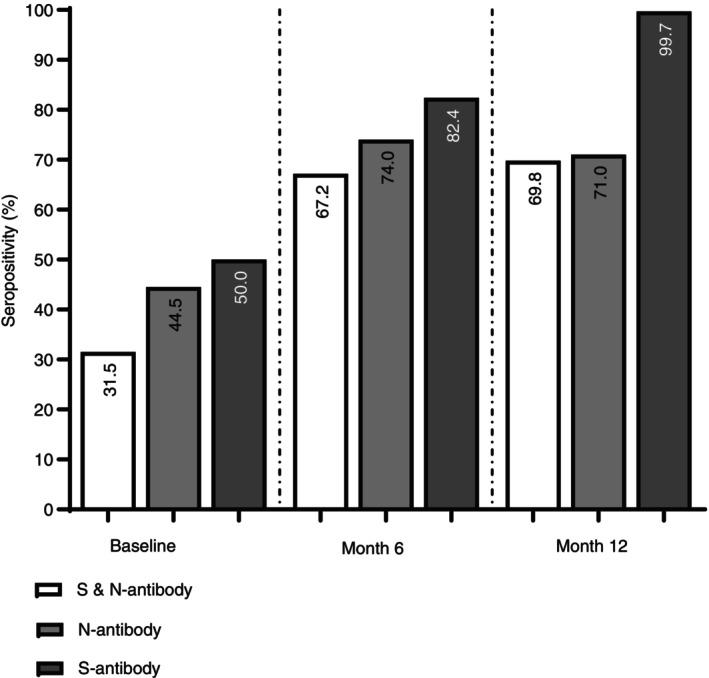
SARS‐CoV‐2 seropositivity across the three timepoints. Bars show the proportion of participants who were spike (S)–only, nucleocapsid (N)–only, and positive for both antigens (S + N) at each time point. Only participants who were unvaccinated at the time of determining serostatus are included.

At baseline (August–November 2021), 491 participants had baseline serology, and 476 (96.9%) were unvaccinated. Of the 476, 63.0% (95% CI, 58.6–67.3) were SARS‐CoV‐2 seropositive (anti‐S or anti‐N). 238 (50.0%) participants were positive for S‐protein antibody, 212 (44.5%) were positive for N‐protein antibody and 150 (31.5%) were positive for both (Figure 2).

At Month 6 (February–May 2022), 402 participants had serology samples and 393 (97.8%) were unvaccinated. SARS‐CoV‐2 seropositivity (anti‐S or anti‐N) increased to 89.3% (95% CI 85.8, 92.0%). 324 (82.4%) were anti‐S positive, 291 (74.0%) were anti‐N positive and 264 (67.2%) were positive for both.

At Month 12 (August–December 2022), 407 participants had serology samples, and 255 (62.7%) were both unvaccinated at enrolment and during follow‐up that occurred between Month 6 and 12 time points. Among the 255, SARS‐CoV‐2 seropositivity had risen to 94.9% (95% CI 91.4, 97.0), with 239 (99.7%) positive for S‐protein antibody, 181 (71.0%) positive for N‐protein antibody and 178 (69.8%) positive for both.

Table 2 summarises unadjusted associations between malaria exposure variables and SARS‐CoV‐2 seropositivity at each time point, alongside other participant characteristics assessed as potential confounders. For the malaria exposures of interest, none of the variables, namely, number of malaria episodes, clinical malaria, asymptomatic malaria and malaria antibody levels, were crudely associated with seropositivity at any timepoint (Table 2).

**TABLE 2:** SARS‐CoV‐2 seropositivity at baseline, Month 6, and Month 12.

Characteristic	Categories	Timepoint = Baseline (*N* = 476)			Timepoint = Month 6 (*N* = 393)			Timepoint = Month 12 (*N* = 255)		
		% sero‐positive	OR (95% CI)^a^	*p*	% sero‐positive	OR (95% CI)^a^	*p*	% sero‐positive	OR (95% CI)^a^	*p*
Age (years)	15	58.1%	1	0.69	88.2%	1	0.29	94.7%	1	0.19
	16	64.0%	1.29 (0.72, 2.31)		90.3%	1.24 (0.46, 3.40)		98.1%	2.92 (0.40, 21.47)	
	17	64.3%	1.30 (0.73, 2.32)		90.2%	1.22 (0.46, 3.29)		92.2%	0.65 (0.13, 3.22)	
	18	53.3%	0.83 (0.27, 2.56)		71.4%	0.33 (0.08, 1.41)		87.5%	0.39 (0.03, 4.90)	
Sex	Male	60.2%	1	0.13	88.2%	1	0.44	92.7%	1	0.08
	Female	66.8%	1.33 (0.91, 1.95)		90.6%	1.29 (0.67, 2.47)		97.5%	3.02 (0.81, 11.24)	
BCG strain at birth	Denmark	46.0%	0.43 (0.21, 0.90)	0.08	96.7%	5.85 (0.76, 45.26)	**0.02**	100%	N/A	0.09
	Russia	63.1%	0.87 (0.57, 1.33)		91.7%	2.22 (1.13, 4.36)		96.2%	2.66 (0.84, 8.42)	
	Bulgaria	66.2%	1		83.2%	1		90.6%	1	
BCG Scar at enrolment	Absent	65.5%	1	0.29	90.5%	1	0.48	96.5%	1	0.29
	Present	60.8%	0.82 (0.56, 1.19)		88.3%	0.79 (0.41, 1.52)		93.6%	0.53 (0.16, 1.77)	
BCG revaccination status at age 13–17 years	No	58.3%	1	0.23	89.1%	1	0.47	88.9%	1	**0.03**
	Yes	65.2%	1.34 (0.83, 2.18)		86.0%	0.75 (0.34, 1.65)		97.4%	4.69 (0.96, 22.87)	
Original EMaBS trial intervention 1 for the mothers during pregnancy	Placebo	61.2%	1	0.41	89.3%	1	0.99	92.9%	1	0.14
	Albendazole	64.9%	1.17 (0.81, 1.70)		89.3%	0.99 (0.52, 1.89)		96.9%	2.40 (0.72, 8.02)	
Original EMaBS trial intervention 2 for the mothers during pregnancy	Placebo	63.2%	1	0.95	87.0%	1	0.13	96.1%	1	0.38
	Praziquantel	62.9%	0.99 (0.68, 1.43)		91.7%	1.65 (0.86, 3.19)		93.7%	0.60 (0.19, 1.90)	
EMaBS trial interventions for the child from 15 months to 5 years	Placebo	59.9%	1	0.18	88.8%	1	0.78	95.0%	1	0.92
	Albendazole	65.9%	1.29 (0.89, 1.88)		89.7%	1.10 (0.58, 2.08)		94.7%	0.95 (0.31, 2.90)	
History of clinically diagnosed allergy during infancy or childhood	No	65.8%	1	**0.03**	90.8%	1	0.10	94.7%	1	0.81
	Yes	54.1%	0.61 (0.40, 0.94)		84.4%	0.55 (0.28, 1.10)		95.5%	1.17 (0.31, 4.40)	
Infection exposure										
Any helminth infection at ages 1–5 and 9 years	No	62.2%	1	0.54	88.0%	1	0.13	94.2%	1	0.35
	Yes	65.3%	1.14 (0.75, 1.76)		93.1%	1.86 (0.80, 4.32)		97.0%	1.98 (0.43, 9.16)	
Any asymptomatic malaria infection at ages 1–5 and 9 years	No	62.3%	1	0.36	89.8%	1	0.47	95.0%	1	0.83
	Yes	68.4%	1.31 (0.73, 2.37)		86.3%	0.72 (0.30, 1.71)		94.1%	0.84 (0.18, 3.96)	
Any clinical malaria episode from birth to 10 years	No	59.8%	1	0.25	86.8%	1	0.20	94.9%	1	0.97
	Yes	65.1%	1.25 (0.86, 1.83)		90.9%	1.53 (0.80, 2.90)		94.9%	1.02 (0.32, 3.21)	
Clinical malaria episodes from birth to 10 years	None	59.8%	1	0.33*	86.8%	1	0.51*	94.9%	1	0.56*
	1 episode	66.0%	1.30 (0.78, 2.18)		90.1%	1.39 (0.58, 3.32)		92.5%	0.67 (0.17, 2.593)	
	2–4 episodes	63.6%	1.18 (0.73, 1.89)		95.6%	3.32 (1.10, 10.05)		95.7%	1.21 (0.28, 5.26)	
	> 4 episodes	66.2%	1.32 (0.75, 2.32)		85.7%	0.92 (0.40, 2.08)		97.1%	1.85 (0.21, 16.39)	
Kaposi's sarcoma–associated herpesvirus infection at 5 years	No	60.6%	1	0.48	88.1%	1	0.67	91.9%	1	0.16
	Yes	64.0%	1.15 (0.78, 1.70)		89.5%	1.16 (0.60, 2.24)		96.2%	2.25 (0.71, 7.10)	
History of lower respiratory tract infection from birth to 10 years	No	61.8%	1	0.44	89.1%	1	0.83	95.0%	1	0.90
	Yes	65.3%	1.16 (0.79, 1.72)		89.8%	1.08 (0.55, 2.13)		94.7%	0.93 (0.30, 2.93)	
Age of CMV infection	Below 2 years	60.1%	1	0.97	89.1%	1	0.73	95.6%	1	0.12
	2 years and above	60.3%	1.01 (0.56, 1.83)		90.9%	1.22 (0.39, 3.77)		87.5%	0.32 (0.08, 1.29)	
Age of HSV infection	Below 2 years	60.4%	1	0.73	91.9%	1	0.36	96.8%	1	0.29
	2 years and above	62.5%	1.09 (0.65, 1.85)		88.1%	0.65 (0.26, 1.64)		92.7%	0.42 (0.08, 2.25)	
Age of norovirus infection	Below 2 years	64.1%	1	0.27	87.0%	1	0.89	94.0%	N/A	N/A
	2 years and above	53.3%	0.64 (0.29, 1.41)		88.0%	1.09 (0.30, 4.05)		100%		
Contact with suspected or confirmed COVID‐19 case prior to enrolment	No	62.7%	1	0.57	89.4%	1	0.83	94.6%	N/A	N/A
	Yes	67.7%	1.25 (0.57, 2.72)		88.0%	0.87 (0.25, 3.04)		100%		
	Yes	86.7%	3.81 (0.85, 17.1)		100%			100%		
Social factors (%, *n*)										
Socioeconomic status at enrolment	Poorest	65.3%	1	0.11	90.8%	1	0.42	95.2%	1	0.09
	Poor	54.9%	0.65 (0.39, 1.09)		92.2%	1.19 (0.45, 3.13)		98.6%	3.56 (0.36, 35.1)	
	Moderate	69.5%	1.21 (0.69, 2.13)		88.2%	0.76 (0.29, 1.96)		96.3%	1.32 (0.21, 8.22)	
	Wealthiest	65.2%	1.0 (0.58, 1.72)		85.2%	0.58 (0.24, 1.44)		89.1%	0.41 (0.10, 1.68)	
Highest level of participant's education	Primary	57.7%	1	0.26	86.1%	1	0.33	93.9%	1	0.72
	Secondary	64.2%	1.32 (0.82, 2.12)		90.0%	1.46 (0.68, 3.12)		95.2%	1.28 (0.34, 4.83)	
Maternal tribe	Central	62.7%	1	0.83	88.2%	1	0.70	95.1%	1	0.95
	Western	62.2%	0.98 (0.58, 1.65)		90.2%	1.23 (0.48, 3.10)		93.0%	0.69 (0.17, 2.71)	
	Eastern	61.7%	0.96 (0.51, 1.81)		92.5%	1.65 (0.48, 5.69)		95.8%	1.19 (0.14, 9.93)	
	Northern	63.1%	1.02 (0.51, 2.06)		94.1%	2.14 (0.49, 9.40)		94.7%	0.93 (0.11, 7.86)	
	Others	76.5%	1.94 (0.62, 6.08)		83.3%	0.69 (0.14, 3.120)		100%	N/A	
Maternal occupation risk exposure at enrolment	High risk	59.8%	1	0.05	89.4%	1	0.51	94.8%	1	
	Moderate risk	68.4%	1.45 (0.84, 2.51)		94.6%	2.09 (0.60, 7.25)		94.6%	0.96 (0.19, 4.81)	0.92
	Low risk	72.2%	1.75 (0.81, 3.78)		84.4%	0.64 (0.22, 1.83)		100%	N/A	
	Unemployed	48.8%	0.64 (0.33, 1.23)		85.3%	0.69 (0.24, 1.95)		96.2%	1.36 (0.16, 11.6)	
	Others	71.4%	1.68 (0.96, 2.93)		88.9%	0.95 (0.38, 2.33)		92.5%	0.67 (0.17, 2.74)	

*Note:* Participants who had been vaccinated prior to enrolment were excluded from all analyses; participants who had been vaccinated between Months 6 and 12 were excluded from Month 12 analysis. Bold values indicate *p*‐values that are statistically significant.

^a^ These were generated from multivariable logistic regression.

* Test for trend *p*‐value.

Among other covariates, participants with a history of allergy were more likely to be seropositive at baseline (*p* = 0.03). At Month 6, those who had received the Danish BCG vaccine strain at birth were more likely to be seropositive (*p* = 0.02), while at Month 12, participants who had received BCG revaccination showed higher odds of seropositivity (*p* = 0.03) (Table 2).

### 3.3 SARS‐CoV‐2 Antibody Concentrations and Crude Associations

Table S1 presents crude associations of malaria exposure variables and potential confounders with SARS‐CoV‐2 S‐protein antibody concentrations at each time point. For the malaria exposures of interest, none of the variables were associated with S‐antibody concentrations at any time point. Among the covariates, participants who reported contact with a suspected or confirmed COVID‐19 case (*p* = 0.04) had higher S‐antibody concentrations at baseline. At Month 6, females (*p* = 0.02) and participants with a higher education level (*p* = 0.001) had significantly higher S‐antibody concentrations. Only maternal praziquantel exposure during pregnancy was associated with lower S‐antibody concentrations at Month 12 (*p* = 0.04).

Table S2 presents crude associations of malaria exposure variables and potential confounders with N‐protein antibody concentrations at each time point. For the malaria exposures of interest, only clinical malaria was associated with higher N‐antibody concentrations at Month 6 (*p* = 0.04). Among other covariates, older age (*p* = 0.04) was associated with lower N‐antibody concentrations at baseline. At Month 6, participants who had received albendazole between 15 months and 5 years in the EMaBS trial had higher N‐antibody concentrations compared with those who received a placebo (*p* = 0.02), and those with a history of clinically diagnosed allergy also had lower concentrations (*p* = 0.02). No covariate showed a significant association with N‐antibody concentrations at Month 12.

### 3.4 Adjusted Association Between Malaria Exposures and SARS‐CoV‐2 Seropositivity

Following adjustment for confounders, there was no consistent evidence that malaria exposures were associated with SARS‐CoV‐2 seropositivity across the study time points (Table 3). Clinical malaria, asymptomatic malaria parasitaemia and malaria episodes showed no statistically significant association with seropositivity at baseline, 6, or 12 months.

**TABLE 3:** Adjusted odds ratios for the association between malaria exposures and SARS‐CoV‐2 seropositivity at baseline, 6 months and 12 months.

Main malaria exposures		Month 0 (*N* = 476)		Month 6 (*N* = 393)		Month 12 (*N* = 255)	
		Adjusted OR (CI)	*p*	Adjusted OR (CI)	*p*	Adjusted OR (CI)	*p*
Clinical malaria from birth to 10 years		1.29 (0.86, 1.94)	0.22	1.22 (0.61, 2.44)	0.58	0.93 (0.28, 3.11)	0.90
Asymptomatic malaria at ages 1–5 and 9 years		1.23 (0.66, 2.30)	0.51	0.55 (0.22, 1.37)	0.22	0.63 (0.12, 3.38)	0.61
Clinical malaria episodes from birth to 10 years	None	REF	0.68	REF	0.11	REF	0.79
	1 episode	1.28 (0.74, 2.22)		1.18 (0.46, 3.06)		0.63 (0.15, 2.66)	
	2–4 episodes	1.31 (0.79, 2.17)		2.61 (0.84, 8.17)		1.04 (0.23, 4.74)	
	> 4 episodes	1.26 (0.69, 2.33)		0.62 (0.25, 1.53)		1.93 (0.19, 19.75)	
Malaria serology at age 13–17 years	PfMSP‐2	1.51 (0.57, 4.05)	0.40	4.74 (0.71, 31.51)	0.10	1.86 (0.09, 40.90)	0.69
	PfAMA‐1	0.80 (0.55, 1.17)	0.24	0.75 (0.39, 1.47)	0.40	1.41 (0.57, 3.49)	0.46

*Note:* All values generated by multivariable logistic regression and adjusted for age, sex, socioeconomic status, history of clinically diagnosed allergy, and BCG strain at birth. Participants who had been vaccinated prior to enrolment were excluded from all analyses; participants who had been vaccinated between Months 6 and 12 were excluded from Month 12 analysis.

Malaria serology showed no significant association with SARS‐CoV‐2 seropositivity, with neither PfMSP‐2 nor PfAMA‐1 antibody levels demonstrating effects at baseline, Month 6, or Month 12 (Table 3).

In sensitivity analyses restricted to participants with anti‐nucleocapsid IgG seropositivity and excluding all vaccinated individuals, the direction and magnitude of associations between malaria exposures and SARS‐CoV‐2 seropositivity were similar. No malaria exposure variable showed a consistent association with seropositivity across time points (Table S3).

### 3.5 Adjusted Association Between Malaria Exposures and SARS‐CoV‐2 Antibody Concentrations

Similarly, confounder‐adjusted analysis of log_10_‐transformed SARS‐CoV‐2 antibody levels provided no consistent evidence of an effect of malaria exposures. For S‐protein antibodies, no malaria exposures showed any significant association at any time point (Table 4). For N‐protein antibodies, clinical malaria was associated with modestly higher antibody concentration at 6 months (mean difference 0.18, 95% CI 0.02, 033; *p* = 0.01), and PfMSP‐2 antibody titres were associated with higher antibody concentration at baseline (mean difference 0.36 95% CI 0.02, 0.71; *p* = 0.04), but these associations were not consistent across timepoints (Table 5).

**TABLE 4:** Mean differences in SARS‐CoV‐2 log S‐Protein antibody levels by malaria exposures at baseline, 6 months, and 12 months.

Main malaria exposures		Month 0 (*N* = 476)		Month 6 (*N* = 393)		Month 12 (*N* = 255)	
		Mean SARS‐CoV‐2 log antibody difference (95% CI)	*p*	Mean SARS‐CoV‐2 log antibody difference (95% CI)	*p*	Mean SARS‐CoV‐2 log antibody difference (95% CI)	*p*
Clinical malaria from birth to 10 years		−0.02 (−0.26, 0.21)	0.85	0.16 (−0.04, 0.35)	0.12	−0.07 (−0.28, 0.14)	0.54
Asymptomatic malaria at ages 1–5 and 9 years		0.04 (−0.31, 0.39)	0.78	0.08 (−0.21, 0.37)	0.57	0.08 (−0.21, 0.37)	0.60
Clinical malaria episodes from birth to 10 years	None	REF	0.39	REF	0.27	REF	0.13
	1 episode	0.15 (−0.16, 0.46)		0.11 (−0.16, 0.37)		−0.25 (−0.52, 0.02)	
	2–4 episodes	−0.14 (−0.43, 0.15)		0.25 (−0.002, 0.51)		−0.01 (−0.26, 0.24)	
	> 4 episodes	−0.06 (−0.41, 0.28)		0.09 (−0.19, 0.37)		0.12 (−0.20, 0.44)	
Malaria serology at age 13–17 years	PfMSP‐2	0.27 (−0.29, 0.83)	0.34	0.33 (−0.17, 0.83)	0.20	0.10 (−0.44, 0.64)	0.71
	PfAMA‐1	−0.06 (−0.27, 0.16)	0.58	−0.05 (−0.24, 0.14)	0.60	0.03 (−0.18, 0.23)	0.80

*Note:* All values generated by multivariable linear regression and adjusted for age, sex, EMaBS mother intervention with praziquantel, contact with a COVID‐19 case, participant's education, area of current residence, and history of clinically diagnosed allergy. Participants who had been vaccinated prior to enrolment were excluded from all analyses; participants who had been vaccinated between Months 6 and 12 were excluded from Month 12 analysis.

**TABLE 5:** Mean differences in SARS‐CoV‐2 log N‐Protein antibody levels by malaria exposures at baseline, 6 months, and 12 months.

Main malaria exposures		Month 0 (*N* = 476)		Month 6 (*N* = 393)		Month 12 (*N* = 255)	
		Mean SARS‐CoV‐2 log antibody difference (95% CI)	*p*	Mean SARS‐CoV‐2 log antibody difference (95% CI)	*p*	Mean SARS‐CoV‐2 log antibody difference (95% CI)	*p*
Clinical malaria from birth to 10 years		0.08 (−0.07, 0.23)	0.23	0.18 (0.02, 0.33)	**0.01**	−0.05 (−0.24, 0.15)	0.63
Asymptomatic malaria at ages 1–5 and 9 years		−0.13 (−0.36, 0.09)	0.24	−0.05 (−0.27, 0.17)	0.68	−0.03 (−0.31, 0.24)	0.82
Clinical malaria episodes from birth to 10 years	None	REF	0.76	REF	0.12	REF	0.33
	1 episode	0.08 (−0.12, 0.28)		0.22 (0.02, 0.42)		−0.13 (−0.39, 0.12)	
	2–4 episodes	0.08 (−0.11, 0.27)		0.18 (−0.02, 0.37)		−0.07 (−0.31, 0.16)	
	> 4 episodes	0.09 (−0.13, 0.31)		0.13 (−0.09, 0.34)		0.15 (−0.15, 0.45)	
Malaria serology at age 13–17 years	PfMSP‐2	0.36 (0.02, 0.71)	**0.04**	0.32 (−0.05, 0.70)	0.09	−0.01 (−0.47, 0.45)	0.96
	PfAMA‐1	0.002 (−0.13, 0.13)	0.97	0.02 (−0.12, 0.16)	0.76	0.03 (−0.14, 0.21)	0.71

*Note:* All values generated by multivariable linear regression and adjusted for Age, sex, EMaBS intervention (babies), maternal tribe, occupation exposure risk, EMaBS maternal PZQ intervention, history of allergy and current residence. Participants who had been vaccinated prior to enrolment were excluded from all analyses; participants who had been vaccinated between Months 6 and 12 were excluded from Month 12 analysis. Bold values indicate *p*‐values that are statistically significant.

### 3.6 COVID‐19 Disease Incidence

Across the study, 4847 symptom‐monitoring phone calls were conducted to assess recent COVID‐19 symptoms. At the time of the call, 904 (19%) reported at least one current symptom. The most frequently reported symptoms were runny nose (610 calls, 13%), cough (404, 8%) and headache (179, 4%); all other symptoms were reported in < 2% of calls. Among calls with a current symptom reported (*n* = 904), 514 (57%) reported a single symptom, 271 (30%) reported 2 symptoms, 76 (8%) reported 3 symptoms and 43 (5%) reported 4 or more symptoms. The median number of successful calls per participant was 10 (IQR 7–12) out of a maximum of 24 possible calls over 12 months. Despite frequent symptom surveillance, no PCR‐confirmed SARS‐CoV‐2 infections were reported, precluding analysis of associations between malaria exposure and clinical COVID‐19 disease incidence or severity in this age group.

## 4 Discussion

In this longitudinal study of Ugandan adolescents nested within a birth cohort, we observed a rapid increase in SARS‐CoV‐2 seropositivity (as a proxy for susceptibility) from 63.7% at baseline in late 2021 to 94.9% at 12 months. Although mild respiratory symptoms were fairly common, no PCR‐confirmed infections were reported. This trajectory is consistent with findings from other African settings where serological surveys have demonstrated widespread infection despite low numbers of confirmed cases and deaths [5, 30, 31]. The absence of confirmed PCR‐positive cases means we cannot draw firm conclusions regarding the factors associated with COVID‐19 disease presentation in this young age group.

The substantial rise in seropositivity prior to the rollout of the national vaccination campaign among adolescents demonstrates community transmission. This pattern aligns with previous evidence indicating that a large proportion of SARS‐CoV‐2 infections in Africa are clinically mild or asymptomatic, contributing to under‐detection by routine surveillance [7, 13]. Such trends are particularly plausible in an adolescent population and during the Omicron‐dominated period, when infection was associated with milder disease and higher rates of asymptomatic infection [32].

Our study adds to previous evidence by focusing on adolescents with well‐characterised life‐course malaria exposures, providing a unique opportunity to examine whether life‐course malaria modifies susceptibility to SARS‐CoV‐2 infection, as reflected by differences in seroconversion across follow‐up. We found no consistent evidence that life‐course malaria exposure, whether measured as clinical episodes, asymptomatic parasitaemia or serological markers, was associated with SARS‐CoV‐2 seropositivity or antibody concentrations. Associations observed with malaria antibody titres (PfMSP‐2 and PfAMA‐1) were inconsistent across timepoints. Overall, our findings do not support a major role of life‐course malaria exposure in shaping susceptibility to SARS‐CoV‐2 infection in adolescents in Africa. These results are consistent with findings from another longitudinal cohort study, which did not demonstrate any relationship between recent malaria and subsequent response to SARS‐CoV‐2 among participants with a median age of 20 years (IQR 9–37) [16]. In contrast, emerging evidence from a Kenyan longitudinal cohort study reports durable immunosuppressive effects of early childhood malaria on antibody responses to unrelated pathogens and vaccines, suggesting that malaria‐related immune modulation may be detectable for some immune endpoints even when associations with SARS‐CoV‐2 serological outcomes are not apparent (preprint) [33].

Due to no observed symptomatic COVID‐19 disease incidence, we were unable to investigate associations between malaria and COVID‐19 disease presentation in this cohort. By contrast, a Ugandan hospital‐based study reported that low previous *P. falciparum* exposure compared to high previous exposure was associated with more severe COVID‐19 manifestations [34]. Similarly, a study from Ghana found that higher *P. falciparum* exposure was associated with reduced severity of SARS‐CoV‐2 infection among individuals aged between 1 and 65 years living in malaria‐endemic regions [15]. The young age of participants and the timing of our study during the Omicron wave, when COVID‐19 disease was generally milder, may partly explain these contrasting outcomes.

Possible mechanisms have been proposed to explain potential interactions between malaria and SARS‐CoV‐2. One hypothesis relates primarily to disease severity: malaria has been suggested to induce cross‐reactive T‐cell responses that dampen proinflammatory cytokine production, potentially reducing the severity of COVID‐19 while still allowing viral transmission [13, 15]. A second distinct hypothesis concerns susceptibility to infection, whereby shared immune‐dominant epitopes between the SARS‐CoV‐2 spike protein and the *Plasmodium falciparum* thrombospondin‐related anonymous protein (TRAP), a protein essential for sporozoite motility and hepatocyte invasion, have been proposed to mediate cross‐reactive immune responses [35, 36]. More recent experimental studies have demonstrated that antibodies elicited during malaria infection can bind to the SARS‐CoV‐2 spike protein, primarily through the recognition of sialylated N‐linked glycans, resulting in substantial cross‐reactivity at the binding level [37, 38]. However, functional assays have demonstrated that these cross‐reactive antibodies do not neutralise SARS‐CoV‐2 in vitro, suggesting possible limited relevance for protection against infection [38]. In our study, which assessed antibody binding rather than functional activity, we found no consistent evidence that malaria exposure influenced susceptibility to SARS‐CoV‐2 infection in adolescents. Future studies could extend these findings by evaluating antibody‐dependent cellular cytotoxicity and by examining clinical endpoints across different age groups.

Beyond malaria, from crude analyses done to inform selection of confounders for associations between malaria and SARS‐CoV‐2 infection, we identified other correlates of SARS‐CoV‐2 serological outcomes. Baseline seropositivity was negatively associated with a history of allergy. Although BCG vaccination has been hypothesised to confer protection against SARS‐CoV‐2 infection [39], our findings do not support a protective effect. Instead, at Month 6, receipt of the Danish BCG strain at birth, known to induce stronger immune responses and more pronounced scarring than other strains [40], was associated with a higher odds of seropositivity, while at Month 12, BCG revaccination was also associated with higher seropositivity. Together, these observations suggest a pattern in which more immunogenic BCG exposures were associated with an increased likelihood of serological evidence of infection, rather than protection. However, these associations were imprecise, with wide CIs, not adjusted for confounders, not consistent across timepoints and should therefore be interpreted with caution. Sociodemographic factors, including education, maternal occupation, and place of residence (urban vs. rural), were crudely associated with higher SARS‐CoV‐2 antibody concentrations at different timepoints, highlighting the multifactorial drivers of exposure and immunity in African settings where infections, vaccination and social determinants of health intersect.

Strengths of this study include its foundation in a well‐established birth cohort with long‐term prospective data, integration of randomised trial exposures and longitudinal follow‐up during the COVID‐19 pandemic. Measurement of both spike and nucleocapsid‐specific antibodies, supported by the documentation of vaccination data, enabled us to distinguish infection from vaccine‐induced responses. The use of a quantitative in‐house ELISA assay, alongside pre‐pandemic samples to establish seropositivity cutoffs, provided additional rigour.

Nonetheless, several limitations should be acknowledged. First, malaria antibody assays were available only for a subset of participants who took part in the POPVAC C trial, reducing statistical power for some analyses. Second, malaria exposure may have been misclassified, as infections could have occurred between scheduled assessments or outside documented clinical encounters; however, such misclassification is likely to be non‐differential with respect to SARS‐CoV‐2 outcome and may have biased estimates towards the null. Third, the effective sample size at Month 12 was smaller because participants who reported COVID‐19 vaccination at baseline or during follow‐up were excluded from Month‐12 statistical analyses, further limiting the precision of estimates at this time point. Fourth, reliance on serological outcomes meant we were unable to assess cellular immune responses, which are known to play an important role in protection against SARS‐CoV‐2 infection and disease [15]. Fifth, SARS‐CoV‐2 antibody titres decline over time following natural infection [41, 42], which may have led to an underestimation of cumulative exposure; however, follow‐up was short and IgG responses, which persist longer than other immunoglobulin classes, were measured. Sixth, classification of SARS‐CoV‐2 serostatus based on spike antibodies may reflect unreported vaccination or prior infection with waned anti‐nucleocapsid responses. To address this potential misclassification, we conducted sensitivity analyses restricted to anti‐nucleocapsid seropositivity. These analyses yielded findings consistent with the primary results. Additionally, we excluded participants who reported vaccination at baseline and during study follow‐up in all statistical analyses subsequent to vaccination. Sixth, PCR testing was managed outside the study, limiting our ability to assess clinical COVID‐19 incidence or severity in this age group. Finally, as the study was restricted to adolescents, the findings may not be generalisable to other age groups.

## 5 Conclusion

We found no consistent evidence that life‐course malaria exposure was associated with SARS‐CoV‐2 infection as measured by serology in adolescents from a malaria‐endemic setting. The high levels of SARS‐CoV‐2 seropositivity observed, despite low reported case numbers, highlight an unresolved discrepancy that may reflect unmeasured host or environmental factors influencing infection dynamics in African populations and warrants further investigation.

## Funding

This research was funded by the Science for Africa Foundation, Grant Ref: GCA/SARSCov2‐2‐20‐002 for the CoHost study. Additional funding was obtained from the National Institute for Health and Care Research (NIHR) under its [‘NIHR Global Health Research Group on Vaccines for vulnerable people in Africa (VAnguard)’] (grant reference number: NIHR134531) using UK aid from the UK Government to support global health research. The views expressed in this publication are those of the authors and not necessarily those of the NIHR or the UK government. This work was also supported by the LSHTM ITD Faculty Equity, Diversity and Inclusion (EDI) Award, and a PhD Scholarship from the MRC/UVRI & LSHTM Uganda Research Unit (MUL/HR/22/0472/7). The POPVAC programme was funded by the UK Medical Research Council (MRC) (MR/R02118X/1 and MC_PC_21034), jointly funded with the UK Foreign, Commonwealth and Development Office (FCDO) under the MRC/FCDO Concordat agreement and as part of the EDCTP2 programme supported by the European Union. The funders had no role in study design, data collection and analysis, decision to publish or preparation of the manuscript. The work was conducted at the MRC/UVRI and LSHTM Uganda Research Unit, which is jointly funded by the UK Medical Research Council (MRC), part of UK Research and Innovation (UKRI) and the UK Foreign, Commonwealth and Development Office (FCDO) under the MRC/FCDO Concordat agreement and is also part of the EDCTP2 programme supported by the European Union.

## Ethics Statement

Ethical approvals were obtained from the Uganda Virus Research Institute (GC/127/35) and the London School of Hygiene and Tropical Medicine (Ref: 8811‐8) Research and Ethics Committees and the Uganda National Council for Science and Technology (UNCST) (Reference no. MV 625).

## Consent

The authors have nothing to report.

## Conflicts of Interest

The authors declare no conflicts of interest.

## Supporting information


**Appendix S1:** Detection of IgG antibodies to SARS‐CoV‐2 spike–and nucleocapsid‐protein antigens in human plasma samples.

**Table S1:** Associations of characteristics with SARS‐CoV‐2 S‐protein antibody concentration across the three timepoints.
**Table S2:** Associations of characteristics with SARS‐CoV‐2 N‐protein antibody concentration across the three timepoints.
**Table S3:** Associations between malaria exposures and SARS‐CoV‐2 seropositivity restricted to anti‐nucleocapsid‐positive participants.

## Data Availability

The de‐identified individual participant data that underlie the results reported in this Article are stored in a non‐publicly available repository (London School of Hygiene and Tropical Medicine [LSHTM] Data Compass), together with a data dictionary accessible via https://doi.org/10.17037/DATA.00005044. Researchers who would like to access the data may submit a request through LSHTM Data Compass, detailing the data requested, the intended use for the data and evidence of relevant experience and other information to support the request. The request will be reviewed in consultation with the Medical Research Council (MRC), Uganda Virus Research Institute (UVRI) and the LSHTM data management committee, with oversight from the UVRI and LSHTM ethics committees. In line with the MRC policy on data sharing, there will have to be a good reason for turning down a request. Researchers given access to the data will sign data sharing agreements that will restrict the use to answering pre‐specified research questions.
