# Supplementary material for: Life Course Malaria Exposure and SARS‐CoV‐2 Seroepidemiology in Ugandan Adolescents: A Longitudinal Study Nested in a Birth Cohort
Source: Trop Med Int Health. 2026 Apr 23;31(8):1012–27. doi: 10.1111/tmi.70148 (PMC13432706; doi:10.1111/tmi.70148)
Supplement: Supplementary file 1 — Appendix S1: Detection of IgG antibodies to SARS‐CoV‐2 spike–and nucleocapsid‐protein antigens in human plasma samples. [file TMI-31-1012-s002.docx]

Supplementary appendix 1:

Detection of IgG antibodies to SARS-CoV-2 spike–and nucleocapsid-protein antigens in human plasma samples

Specific IgG antibodies to the SARS-CoV-2 Spike (S) protein and nucleocapsid (N) protein were measured using an in-house IgG ELISA. The S antigen was the recombinant perfusion-stabilised ectodomain of the SARS-CoV-2 spike glycoprotein, Wuhan-Hu-1(GenPept: QHD43416), and the N antigen was a recombinant SARS-CoV-2 nucleocapsid protein. Both antigens were kindly provided by ATCC-BEI Resources (Manassas, VA, USA). Medium-binding 96-well plates were coated overnight with 50μl per well of S-antigen/N-antigen at a coating concentration of 1.25μg/ml (S-protein) and 1 μg/ml (N-protein). The plates were washed 4 times with phosphate-buffered saline (PBS 1X)-Tween 20 (0.05%) (Sigma-Aldrich, UK) solution and blocked with 200μl per well of 5% skimmed milk (Sigma-Aldrich, UK) diluted in PBS-Tween 20 (Sigma-Aldrich, UK) and incubated for 1 hour at room temperature (RT). The plates were then washed and incubated for 2 hours at RT with 50μl of test plasma, positive and negative control samples (diluted 1/100 with 1% skimmed milk in PBS-Tween 20). Positive controls for S-protein consisted of Monoclonal Anti-SARS Coronavirus Recombinant Human IgG1 (Clone CR3022 produced in Nicotiana benthamiana Catalog No. NR-53876) obtained from ATCC – BEI resources (Manassas, VA, USA). For the N protein, positive controls were a pool of serum samples from the assay optimisation that showed high optical density (OD) signals to the N protein. Following incubation of test samples, positive and negative control samples, plates were washed, and detection antibody conjugated to goat anti-human IgG-horseradish peroxidase conjugate (Insight Biotechnology, UK), diluted 1/5000 in assay buffer, was added. The plates were incubated for 1 hour at room temperature, washed, and developed by the addition of 100μl of O-Phenylenediamine Dihydrochloride (OPD) substrate (Sigma-Aldrich, UK), and reactions were stopped after 5 minutes with 30μl of 2M sulphuric acid (Fisher Scientific, UK). Optical density (OD) was measured at 450nm using a 96-well ELISA plate reader (BioTek ELx808, USA). A standard curve was generated using purified human IgG (Sigma-Aldrich, UK, Catalogue No. I2511), and OD values were interpolated to derive antibody concentrations expressed as arbitrary ELISA units. To minimise batch effects, all samples were randomised across all plates and done within the same period to ensure uniform experimental conditions and reduce potential variability and spurious observations which could arise from batch-to-batch differences.
