# Supplementary material for: Life Course Malaria Exposure and SARS‐CoV‐2 Seroepidemiology in Ugandan Adolescents: A Longitudinal Study Nested in a Birth Cohort
Source: Trop Med Int Health. 2026 Apr 23;31(8):1012–27. doi: 10.1111/tmi.70148 (PMC13432706; doi:10.1111/tmi.70148)
Supplement: Supplementary file 2 — Table S1: Associations of characteristics with SARS‐CoV‐2 S‐protein antibody concentration across the three timepoints. Table S2: Associations of characteristics with SARS‐CoV‐2 N‐protein antibody concentration across the three timepoints. Table S3: Associations between malaria exposures and SARS‐CoV‐2 seropositivity restricted to anti‐nucleocapsid‐positive participants. [file TMI-31-1012-s001.docx]

**Supplementary Table 1: Associations of characteristics with SARS-CoV-2 S-protein antibody concentration across the three timepoints.**

|  |  | **Timepoint = Baseline^#^** | | | **Timepoint = Month 6^#^** | | | **Timepoint = Month 12^#^** | | |
| --- | --- | --- | --- | --- | --- | --- | --- | --- | --- | --- |
| **Characteristic** | **Categories** | **Mean (SD)**  **SARS-CoV-2 log antibody levels** | **Mean difference**  **(95% CI)** | **P** | **Mean (SD)**  **SARS-CoV-2 log antibody levels** | **Mean difference**  **(95% CI)** | **P** | **Mean (SD)**  **SARS-CoV-2 log antibody levels** | **Mean difference**  **(95% CI)** | **P** |
| Age (years) | 15 | 4.17 (1.22) | - | 0.33 | 5.31 (1.23) | - | 0.74 | 5.54 (1.11) | - | 0.73 |
|  | 16 | 4.35 (1.25) | 0.17 (-0.19, 0.54) |  | 5.38 (0.94) | 0.07 (-0.24, 0.39) |  | 5.74 (0.38) | 0.20 (-0.10, 0.50) |  |
|  | 17 | 4.37 (1.29) | 0.20 (-0.16, 0.56) |  | 5.46 (0.96) | 0.15 (-0.16, 0.46) |  | 5.62 (0.96) | 0.08 (-0.22, 0.38) |  |
|  | 18 | 3.84 (1.53) | -0.33 (-1.05, 0.38) |  | 5.31 (0.93) | 0.003 (-0.59, 0.59) |  | 5.62 (0.94) | 0.08 (-0.53, 0.70) |  |
| Sex | Male | 4.26 (1.32) | - | 0.24 | 5.28 (1.08) | - | **0.02** | 5.60 (0.90) | - | 0.28 |
|  | Female | 4.40 (1.20) | 0.14 (-0.09, 0.37) |  | 5.54 (0.86) | 0.25 (0.06, 0.45) |  | 5.71 (0.65) | 0.11 (-0.09, 0.30) |  |
| BCG strain at birth | Bulgaria | 4.25 (1.33) | - | 0.44 | 5.31 (1.10) | - | 0.42 | 5.50 (1.21) | - | 0.12 |
|  | Russia | 4.38 (1.23) | 0.13 (-0.12, 0.39) |  | 5.45 (0.90) | 0.14 (-0.08, 0.35) |  | 5.72 (0.49) | 0.22 (-0.002, 0.44) |  |
|  | Denmark | 4.16 (1.20) | -0.09 (-0.54, 0.37) |  | 5.48 (0.89) | 0.17 (-0.22, 0.56) |  | 5.75 (0.35) | 0.25 (-0.13, 0.62) |  |
| BCG Scar at enrolment | Absent | 4.34 (1.21) | - | 0.68 | 5.45 (1.03) | - | 0.36 | 5.64 (0.89) | - | 0.81 |
|  | Present | 4.29 (1.32) | -0.05 (-0.28, 0.18) |  | 5.36 (0.96) | -0.09 (-0.29, 0.11) |  | 5.66 (0.72) | 0.02 (-0.18, 0.22) |  |
| BCG revaccination status at age 13–17 years | No | 4.21 (1.21) | - | 0.83 | 5.32 (0.99) | - | 0.44 | 5.49 (1.14) | - | 0.25 |
|  | Yes | 4.24 (1.42) | 0.03 (-0.28, 0.35) |  | 5.20 (1.23) | -0.11 (-0.41, 0.18) |  | 5.67 (0.77) | 0.18 (-0.13, 0.49) |  |
| Original EMaBS trial intervention 1 for the mothers during pregnancy | Placebo | 4.26 (1.25) | - | 0.29 | 5.36 (1.05) | - | 0.44 | 5.59 (0.91) | - | 0.18 |
|  | Albendazole | 4.38 (1.29) | 0.12 (-0.10, 0.35) |  | 5.44 (0.92) | 0.08 (-0.12, 0.27) |  | 5.72 (0.67) | 0.13 (-0.06, 0.33) |  |
| Original EMaBS trial intervention 2 for the mothers during pregnancy | Placebo | 4.32 (1.33) | - | 0.93 | 5.36 (1.06) | - | 0.36 | 5.76 (0.67) | - | **0.04** |
|  | Praziquantel | 4.32 (1.21) | 0.01 (-0.22, 0.24) |  | 5.45 (0.91) | 0.09 (-0.10, 0.29) |  | 5.55 (0.89) | -0.21 (-0.40, -0.01) |  |
| EMaBS trial interventions for the child from 15 months to 5 years | Placebo | 4.26 (1.27) | - | 0.38 | 5.34 (1.06) | - | 0.24 | 5.65 (0.74) | - | 0.99 |
|  | Albendazole | 4.37 (1.27) | 0.10 (-0.13, 0.33) |  | 5.45 (0.92) | 0.11 (-0.08, 0.32) |  | 5.65 (0.85) | -0.001 (-0.20, 0.20) |  |
| History of clinically diagnosed allergy during infancy or childhood | No | 4.37 (1.26) | - | 0.09 | 5.43 (0.97) | - | 0.22 | 5.67 (0.76) | - | 0.51 |
|  | Yes | 4.14 (1.30) | -0.23 (-0.50, 0.04) |  | 5.29 (1.05) | -0.15 (-0.38, 0.09) |  | 5.60 (0.91) | -0.06 (-0.30, 0.15) |  |
| **Infection Exposure** | | | | | | | | | | |
| Any helminth infection at ages 1-5 and 9 years | No | 4.30 (1.28) | - | 0.52 | 5.39 (1.03) | - | 0.64 | 5.62 (0.89) | - | 0.28 |
|  | Yes | 4.38 (1.25) | 0.08 (-0.18, 0.35) |  | 5.44 (0.86) | 0.05 (-0.17, 0.28) |  | 5.75 (0.40) | 0.12 (-0.10, 0.35) |  |
| Any asymptomatic malaria infection at ages 1-5 and 9 years | No | 4.31 (1.29) | - | 0.66 | 5.39 (1.01) | - | 0.47 | 5.64 (0.84) | - | 0.51 |
|  | Yes | 4.39 (1.18) | 0.08 (-0.28, 0.43) |  | 5.49 (0.87) | 0.11 (-0.18, 0.40) |  | 5.74 (0.36) | 0.10 (-0.19, 0.39) |  |
| Any clinical malaria episode from birth to 10 years | No | 4.32 (1.35) | - | 0.96 | 5.28(1.15) | - | 0.06 | 5.70 (0.60) | - | 0.45 |
|  | Yes | 4.32 (1.22) | 0.01 (-0.23, 0.24) |  | 5.47 (0.87) | 0.19(-0.01, 0.39) |  | 5.62(0.90) | -0.08 (-0.28, 0.12) |  |
| Clinical malaria Episodes from birth to 10 years | None | 4.32 (1.35) | - | 0.55^*^ | 5.28 (1.15) | - | 0.13* | 5.70 (0.60) | - | 0.61* |
|  | 1 episode | 4.49 (1.24) | 0.17 (-0.14, 0.49) |  | 5.43 (0.92) | 0.14 (-0.12, 0.41) |  | 5.44 (1.21) | -0.26 (-0.53, 0.01) |  |
|  | 2-4 episodes | 4.22(1.25) | -0.10 (-0.39, 0.19) |  | 5.57 (0.75) | 0.29 (0.03, 0.54) |  | 5.68 (0.79) | -0.03 (-0.27, 0.22) |  |
|  | >4 episodes | 4.28 (1.16) | -0.04 (-0.38, 0.31) |  | 5.40 (0.96) | 0.12 (-0.16, 0.40) |  | 5.80 (0.37) | 0.10 (-0.21, 0.40) |  |
| Kaposis’s – sarcoma associated Herpesvirus infection at 5 years | No | 4.34 (1.24) | - | 0.47 | 5.38 (1.03) | - | 0.78 | 5.54 (1.02) | - | 0.11 |
|  | Yes | 4.25 (1.30) | 0.09 (-0.15, 0.33) |  | 5.42 (0.96) | -0.03 (-0.24, 0.18) |  | 5.72 (0.64) | 0.17 (-0.04, 0.39) |  |
| History of Lower Respiratory Tract Infection from birth to 10 years | No | 4.34 (1.30) | - | 0.70 | 5.36(1.01) | - | 0.25 | 5.62 (0.80) | - | 0.44 |
|  | Yes | 4.29 (1.23) | -0.05 (-0.29, 0.19) |  | 5.48 (0.96) | 0.12 (-0.09, 0.33) |  | 5.70 (0.79) | 0.08 (-0.12, 0.28) |  |
| Age of CMV infection | 1 year and below | 4.14 (1.32) | - | 0.75 | 5.38 (0.94) | - | 0.44 | 5.65 (0.86) | - | 0.21 |
|  | Above 2 years | 4.20 (1.32) | 0.06 (-0.32, 0.45) |  | 5.50 (1.01) | 0.12 (-0.19, 0.44) |  | 5.41 (1.29) | -0.24 (-0.63, 0.14) |  |
| Age of HSV infection | 2 years and below | 4.22 (1.27) | - | 0.72 | 5.42 (0.93) | - | 0.99 | 5.59 (1.08) | - | 0.77 |
|  | Above 3 years | 4.16 (1.32) | -0.06 (-0.39, 0.27) |  | 5.42 (1.01) | 0.002 (-0.26, 0.27) |  | 5.63 (0.68) | 0.05 (-0.26, 0.36) |  |
| Age of norovirus infection | 1 year and below | 4.20 (1.39) | - | 0.59 | 5.34 (1.05) | - | 0.35 | 5.66 (0.81) | - | 0.19 |
|  | Above 2 years | 4.05 (1.24) | -0.15 (-0.68, 0.39) |  | 5.54 (0.77) | 0.21 (-0.23, 0.64) |  | 5.92 (0.19) | 0.25 (-0.13, 0.64) |  |
| Contact with a suspected or confirmed COVID-19 case prior to enrolment | No | 4.29 (1.28) | - | **0.04** | 5.40 (0.99) | - | 0.70 | 5.64 (0.82) | - | 0.33 |
|  | Yes | 4.77 (1.10) | 0.48 (0.02, 0.94) |  | 5.47 (0.99) | 0.08 (-0.32, 0.48) |  | 5.86 (0.25) | 0.22 (-0.22, 0.65) |  |
|  | Yes | 5.57 (1.09) | 1.25 (0.60, 1.91) |  | 5.92 (0.17) | 0.52 (-0.13, 1.17) |  | 5.73 (1.01) | 0.08 (-0.37, 0.53) |  |
| **Social factors** | | | | | | | | | | |
| Socioeconomic status at enrolment | Poorest | 4.39 (1.13) | - | 0.55 | 5.48 (0.88) | - | 0.18 | 5.68 (0.85) | - | 0.13 |
|  | Poor | 4.26 (1.29) | -0.12 (-0.44, 0.19) |  | 5.54 (0.80) | 0.06 (-0.20, 0.33) |  | 5.76 (0.40) | 0.08 (-0.19, 0.35) |  |
|  | Moderate | 4.44 (1.25) | 0.05 (-0.29, 0.38) |  | 5.31 (1.13) | -0.16 (-0.45, 0.12) |  | 5.71 (0.55) | 0.03 (-0.26, 0.32) |  |
|  | Wealthiest | 4.23 (1.38) | -0.16 (-0.49, 0.17) |  | 5.28 (1.11) | -0.20 (-0.48, 0.08) |  | 5.45 (0.17) | -0.23 (-0.51, 0.05) |  |
| Highest Level of Participant’s education | Primary | 4.13 (1.46) | - | 0.13 | 5.06 (1.16) | - | **0.001** | 5.68 (0.66) | - | 0.77 |
|  | Secondary | 4.36 (1.23) | 0.23 (-0.07, 0.53) |  | 5.48 (0.93) | 0.42 (0.17, 0.67) |  | 5.65 (0.83) | -0.04 (-0.29, 0.21) |  |
| Maternal tribe | Central | 4.32 (1.23) | - | 0.88 | 5.40 (0.98) | - | 0.47 | 5.67(0.81) | - | 0.90 |
|  | Western | 4.32 (1.41) | 0.004 (-0.32, 0.33) |  | 5.45 (0.83) | 0.05 (-0.23, 0.33) |  | 5.60 (0.96) | -0.06 (-0.33, 0.21) |  |
|  | Eastern | 4.34 (1.30) | 0.03(-0.36, 0.42) |  | 5.46 (1.18) | 0.06 (-0.27, 0.39) |  | 5.73 (0.48) | 0.07 (-0.27, 0.42) |  |
|  | Northern | 4.45 (1.24) | 0.13 (-0.30, 0.57) |  | 5.42 (1.06) | 0.02 (-0.33, 0.38) |  | 5.60 (0.69) | -0.06 (-0.44, 0.32) |  |
|  | Others | 4.05 (1.5) | -0.27 (-0.89, 0.36) |  | 4.89 (1.16) | -0.52 (-1.09, 0.06) |  | 5.89 (0.20) | 0.23 (-0.42, 0.89) |  |
| Maternal occupation risk exposure at enrolment | High risk | 4.35 (1.27) | - | 0.16 | 5.38 (1.02) | - | 0.23 | 5.68 (0.71) | - | 0.73 |
|  | Moderate risk | 4.30 (1.25) | -0.05 (-0.38, 0.27) |  | 5.60(0.69) | 0.22 (-0.07, 0.51) |  | 5.66 (0.56) | -0.02 (-0.31, 0.28) |  |
|  | Low risk | 4.27 (1.40) | -0.08 (-0.52, 0.37) |  | 5.14 (1.37) | -0.24 (-0.61, 0.13) |  | 5.82 (0.33) | 0.14 (-0.27, 0.55) |  |
|  | Unemployed | 3.90 (1.47) | -0.45 (-0.86, -0.04) |  | 5.27 (1.03) | -0.11 (-0.47, 0.25) |  | 5.59 (1.18) | -0.09 (-0.43, 0.25) |  |
|  | others | 4.51 (1.10) | 0.16 (-0.17, 0.48) |  | 5.48 (0.84) | 0.10 (-0.17, 0.38) |  | 5.53 (1.07) | -0.15 (-0.43, 0.13) |  |
| Area of current Residence | Urban | 4.27 (1.29) | - | 0.06 | 5.42 (0.99) | - | 0.55 | 5.65 (0.83) | - | 0.91 |
|  | Rural | 4.57 (1.18) | 0.30 (-0.009, 0.61) |  | 5.34 (0.96) | -0.08 (-0.35, 0.19) |  | 5.67 (0.58) | 0.01 (-0.26, 0.28) |  |
| COVID-19 self-reported symptoms prior to enrolment | No | 4.31 (1.28) | - | 0.41 | 5.39 (1.0) | - | 0.45 | 5.65 (0.80) | - | 0.49 |
|  | Yes | 4.61 (0.76) | 0.29 (-0.41, 1.00) |  | 5.63(0.55) | 0.24 (-0.38, 0.86) |  | 5.90 (0.28) | 0.25 (-0.46, 0.96) |  |
| Body Mass Index at enrolment | Underweight | 4.14 (1.30) | -0.18 (-0.53, 0.16) | 0.33 | 5.29 (1.22) | -0.13 (-0.42, 0.17) | 0.66 | 5.60 (1.18) | -0.05 (-0.34, 0.24) | 0.72 |
|  | Normal | 4.33 (1.26) | - |  | 5.41 (0.97) | - |  | 5.65 (0.75) | - |  |
|  | Overweight and above | 4.51 (1.33) | 0.19 (-0.21, 0.58) |  | 5.45 (0.78) | 0.04 (-0.30, 0.37) |  | 5.78 (0.35) | 0.13 (-0.24, 0.51) |  |

^#^Generated from simple linear regression. *Test for trend p-value. All participants who had been COVID-19-vaccinated prior to baseline were excluded from the analysis. In addition, participants who received vaccination during the study between months 6 and 12 were excluded from analyses at month 12.

**Supplementary Table 2: Associations of characteristics with SARS-CoV-2 N-protein antibody concentration across the three timepoints.**

|  |  | **Timepoint = Baseline^#^** | | | **Timepoint = Month 6^#^** | | | **Timepoint = Month 12^#^** | | |
| --- | --- | --- | --- | --- | --- | --- | --- | --- | --- | --- |
| **Characteristic** | **Categories** | **Mean (SD)**  **SARS-CoV-2 log antibody levels** | **Mean difference**  **(95% CI)** | **P** | **Mean (SD)**  **SARS-CoV-2 log antibody levels** | **Mean difference**  **(95% CI)** | **P** | **Mean (SD)**  **SARS-CoV-2 log antibody levels** | **Mean difference**  **(95% CI)** | **P** |
| Age (years) | 15 | 3.85 (0.60) | - | **0.04** | 4.47 (0.85) | - | 0.67 | 4.28 (0.92) | - | 0.69 |
|  | 16 | 3.74 (0.82) | -0.11 (-0.33, 0.12) |  | 4.34 (0.74) | -0.13 (-0.37, 0.11) |  | 4.29 (0.66) | 0.003 (-0.27, 0.30) |  |
|  | 17 | 3.83 (0.78) | -0.01 (-0.24, 0.21) |  | 4.36 (0.72) | -0.12 (-0.35, 0.11) |  | 4.21 (0.76) | -0.07 (-0.35, 0.21) |  |
|  | 18 | 3.26 (1.21) | -0.58 (-1.03, -0.14) |  | 4.25 (0.64) | -0.22 (-0.66, 0.22) |  | 3.00 (0.62) | -0.28 (-0.85, 0.29) |  |
| Sex | Male | 3.80 (0.79) | - | 0.64 | 4.32 (0.78) | - | 0.20 | 4.24 (0.78) | - | 0.86 |
|  | Female | 3.76 (0.80) | -0.03 (-0.18, 0.11) |  | 4.41 (0.70) | 0.10 (-0.05, 0.25) |  | 4.26 (0.70) | 0.02 (-0.17, 0.20) |  |
| BCG strain at birth | Bulgaria | 3.74 (0.85) | - | 0.59 | 4.30 (0.80) | - | 0.23 | 4.17 (0.96) | - | 0.40 |
|  | Russia | 3.81 (0.80) | 0.08 (-0.09, 0.24) |  | 4.37 (0.74) | 0.06 (-0.10, 0.23) |  | 4.31 (0.63) | 0.14 (-0.06, 0.35) |  |
|  | Denmark | 3.72 (0.69) | -0.02 (-0.31, 0.27) |  | 4.56 (0.51) | 0.26 (-0.04, 0.55) |  | 4.25 (0.47) | 0.08 (-0.27, 0.43) |  |
| BCG Scar at enrolment | Absent | 3.76 (0.71) | - | 0.76 | 4.34 (0.84) | - | 0.70 | 4.24 (0.78) | - | 0.94 |
|  | Present | 3.79 (0.85) | 0.02 (-0.12, 0.17) |  | 4.37 (0.66) | 0.03 (-0.12, 0.18) |  | 4.25 (0.72) | -0.01 (-0.18, 0.19) |  |
| BCG revaccination status at age 13–17 years | No | 3.84 (0.66) | - | 0.87 | 4.43 (0.71) | - | 0.27 | 4.17 (0.95) | - | 0.17 |
|  | Yes | 3.82 (0.85) | -0.02 (-0.20, 0.16) |  | 4.32 (0.85) | -0.12 (-0.32, 0.09) |  | 4.35 (0.63) | 0.18 (-0.08, 0.44) |  |
| Original EMaBS trial intervention 1 for the mothers during pregnancy | Placebo | 3.76 (0.82) | - | 0.60 | 4.33 (0.80) | - | 0.36 | 4.21 (0.76) | - | 0.39 |
|  | Albendazole | 3.80 (0.77) | 0.04 (-0.10, 0.18) |  | 4.40 (0.69) | 0.07 (-0.08, 0.22) |  | 4.29 (0.72) | 0.08 (-0.10, 0.26) |  |
| Original EMaBS trial intervention 2 for the mothers during pregnancy | Placebo | 3.76 (0.87) | - | 0.58 | 4.29 (0.81) | - | 0.06 | 4.30 (0.75) | - | 0.24 |
|  | Praziquantel | 3.80 (0.71) | 0.04 (-0.10, 0.18) |  | 4.43 (0.66) | 0.14 (-0.01, 0.29) |  | 4.19 (0.74) | -0.11 (-0.29, 0.73) |  |
| EMaBS trial interventions for the child from 15 months to 5 years | Placebo | 3.71 (0.84) | - | 0.07 | 4.27 (0.82) | - | **0.02** | 4.23 (0.70) | - | 0.78 |
|  | Albendazole | 3.84 (0.75) | 0.13 (-0.01, 0.28) |  | 4.44 (0.65) | 0.17 (0.03, 0.32) |  | 4.26 (0.78) | 0.03 (-0.16, 0.21) |  |
| History of clinically diagnosed allergy during infancy or childhood | No | 3.81 (0.77) | - | 0.06 | 4.41 (0.67) | - | **0.02** | 4.25 (0.74) | - | 0.99 |
|  | Yes | 3.65 (0.86) | -0.16 (-0.33, 0.01) |  | 4.20 (0.95) | -0.22 (-0.39, -0.04) |  | 4.25 (0.74) | 0.001 (-0.21, 0.21) |  |
| **Infection Exposure** | | | | | | | | | | |
| Any helminth infection at ages 1-5 and 9 years | No | 3.80 (0.76) | - | 0.27 | 4.37 (0.77) | - | 0.82 | 4.24 (0.75) | - | 0.82 |
|  | Yes | 3.71 (0.88) | -0.09 (-0.25, 0.07) |  | 4.35 (0.67) | -0.02 (-0.19, 0.15) |  | 4.27 (0.71) | 0.02 (-0.18, 0.23) |  |
| Any asymptomatic malaria infection at ages 1-5 and 9 years | No | 3.79 (0.78) | - | 0.30 | 4.36 (0.75) | - | 0.90 | 4.25 (0.76) | - | 0.85 |
|  | Yes | 3.68 (0.88) | -0.12 (-0.34, 0.10) |  | 4.35 (0.72) | -0.01 (-0.23, 0.21) |  | 4.23 (0.63) | -0.03 (-0.30, 0.24) |  |
| Any clinical malaria infection from birth to 10 years | No | 3.74 (0.84) | - | 0.44 | 4.26 (0.88) | - | **0.04** | 4.29 (0.58) | - | 0.44 |
|  | Yes | 3.80 (0.77) | 0.06 (-0.09, 0.21) |  | 4.42 (0.64) | 0.16 (0.01, 0.31) |  | 4.22 (0.82) | -0.07 (-0.26, 0.11) |  |
| Clinical malaria Episodes from birth to 10 years | None | 3.74 (0.84) | - | 0.56* | 4.26 (0.88) | - | 0.20* | 4.29 (0.58) | - | 0.83* |
|  | 1 episode | 3.81 (0.75) | 0.07 (-0.13, 0.27) |  | 4.47 (0.64) | 0.21 (0.004, 0.41) |  | 4.12 (0.97) | -0.17 (-0.42, 0.08) |  |
|  | 2-4 episodes | 3.80 (0.76) | 0.05 (-0.13, 0.24) |  | 4.42 (0.57) | 0.16 (-0.03, 0.35) |  | 4.21 (0.85) | -0.09 (-0.32, 0.14) |  |
|  | >4 episodes | 3.80 (0.80) | 0.05 (-0.16, 0.27) |  | 4.37 (0.73) | 0.10 (-0.11, 0.32) |  | 4.39 (0.43) | 0.10 (-0.18, 0.39) |  |
| Kaposis’s – sarcoma associated Herpesvirus infection at 5 years | No | 3.78 (0.76) | - | 0.53 | 4.37 (0.68) | - | 0.58 | 4.26 (0.73) | - | 0.61 |
|  | Yes | 3.73 (0.87) | 0.05 (-0.10, 0.20) |  | 4.32 (0.83) | 0.04 (-0.11, 0.20) |  | 4.20 (0.80) | 0.05 (-0.15, 0.25) |  |
| History of Lower Respiratory Tract Infection from birth to 10 years | No | 3.78 (0.78) | - | 0.87 | 4.34 (0.75) | - | 0.42 | 4.26 (0.73) | - | 0.84 |
|  | Yes | 3.77 (0.83) | -0.01 (-0.16, 0.14) |  | 4.40 (0.73) | 0.06 (-0.09, 0.22) |  | 4.24 (0.77) | -0.02 (-0.21, 0.17) |  |
| Age of CMV infection | 1 year and below | 3.69 (0.86) | - | 0.89 | 4.38 (0.65) | - | 0.28 | 4.28 (0.78) | - | 0.28 |
|  | Above 2 years | 3.71 (0.95) | 0.02 (-0.24, 0.27) |  | 4.49 (0.69) | 0.12 (-0.10, 0.33) |  | 4.10 (1.02) | -0.18 (-0.52, 0.15) |  |
| Age of HSV infection | 2 years and below | 3.74 (0.83) | - | 0.75 | 4.38 (0.69) | - | 0.25 | 4.32 (0.81) | - | 0.34 |
|  | Above 3 years | 3.70 (0.83) | -0.03 (-0.25, 0.18) |  | 4.48 (0.61) | 0.10 (-0.07, 0.28) |  | 4.19 (0.68) | -0.13 (-0.38, 0.13) |  |
| Age of norovirus infection | 1 year and below | 3.75 (0.83) | - | 0.63 | 4.35 (0.75) | - | 0.47 | 4.18 (0.65) | - | 0.10 |
|  | Above 2 years | 3.67 (0.89) | -0.08 (-0.41, 0.25) |  | 4.24 (0.62) | -0.12 (-0.43, 0.20) |  | 4.45 (0.44) | 0.27 (-0.05, 0.59) |  |
| Contact with suspected or confirmed COVID-19 case prior to enrolment | No | 3.76 (0.80) | - | 0.20 | 4.35 (0.75) | - | 0.28 | 4.25 (0.75) | - | 0.85 |
|  | Yes | 3.96 (0.67) | 0.19 (-0.10, 0.48) |  | 4.52 (0.65) | 0.17 (-0.14, 0.47) |  | 4.28 (0.58) | 0.04 (-0.36, 0.44) |  |
|  | Yes | 3.72 (0.74) | -0.06 (-0.47 (0.35) |  | 4.21 (0.34) | -0.15 (-0.64, 0.34) |  | 4.45 (0.55) | 0.20 (-0.21, 0.61) |  |
| **Social factors** | | | | | | | | | | |
| Socioeconomic status at enrolment | Poorest | 3.85 (0.81) | - | 0.26 | 4.38 (0.61) | - | 0.36 | 4.20 (0.77) | - | 0.22 |
|  | Poor | 3.67 (0.87) | -0.18 (-0.37, 0.02) |  | 4.46 (0.65) | 0.08 (-0.12, 0.28) |  | 4.39 (0.53) | 0.19 (-0.06, 0.44) |  |
|  | Moderate | 3.85 (0.80) | -0.002 (-0.21, 0.21) |  | 4.31 (0.85) | -0.06 (-0.28, 0.15) |  | 4.26 (0.78) | 0.06 (-0.21, 0.33) |  |
|  | Wealthiest | 3.77 (0.68) | -0.08 (-0.29, 0.13) |  | 4.28 (0.87) | -0.09 (-0.31, 0.12) |  | 4.13 (0.87) | -0.07 (-0.33, 0.19) |  |
| Highest Level of Participant’s education | Primary | 3.75 (0.77) | - | 0.66 | 4.29 (0.64) | - | 0.39 | 4.32 (0.69) | - | 0.48 |
|  | Secondary | 3.79 (0.80) | 0.04 (-0.15, 0.23) |  | 4.38 (0.77) | 0.08 (-0.11, 0.28) |  | 4.23 (0.75) | -0.08 (-0.32, 0.15) |  |
| Maternal tribe | Central | 3.76 (0.82) | - | 0.93 | 4.35 (0.72) | - | 0.10 | 4.18 (0.72) | - | 0.44 |
|  | Western | 3.84 (0.77) | 0.08 (-0.12, 0.28) |  | 4.33 (0.74) | -0.02 (0.22, 0.19) |  | 4.35 (0.86) | 0.17 (-0.08, 0.42) |  |
|  | Eastern | 3.83 (0.74) | 0.08 (-0.17, 0.32) |  | 4.49 (0.87) | 0.14 (-0.11, 0.39) |  | 4.36 (0.64) | 0.18 (-0.14, 0.50) |  |
|  | Northern | 3.80 (0.83) | 0.05 (-0.22, 0.32) |  | 4.54 (0.70) | 0.20 (-0.07, 0.46) |  | 4.41 (0.78) | 0.22 (-0.13, 0.58) |  |
|  | Others | 3.77 (0.55) | 0.02 (-0.37, 0.41) |  | 3.91 (0.70) | -0.43 (-0.86, -0.002) |  | 4.38 (0.45) | 0.20 (-0.41, 0.80) |  |
| Maternal occupation risk exposure at enrolment | High risk | 3.74 (0.81) | - | 0.65 | 4.36 (0.73) | - | 0.07 | 4.28 (0.72) | - | 0.16 |
|  | Moderate risk | 3.77 (0.82) | 0.03 (-0.17, 0.24) |  | 4.53 (0.73) | 0.16 (-0.06, 0.38) |  | 4.35 (0.60) | 0.07 (-0.20, 0.34) |  |
|  | Low risk | 3.82 (0.94) | 0.08 (-0.20, 0.36) |  | 4.06 (1.06) | -0.31 (-0.58, -0.03) |  | 4.43 (0.62) | 0.15 (-0.23, 0.52) |  |
|  | Unemployed | 3.84 (0.66) | 0.11 (-0.15, 0.36) |  | 4.28 (0.50) | -0.09 (-0.36, 0.18) |  | 4.18 (1.00) | -0.10 (-0.42, 0.21) |  |
|  | others | 3.89 (0.73) | 0.15 (-0.05, 0.36) |  | 4.39 (0.69) | 0.02 (-0.19, 0.23) |  | 4.00 (0.76) | -0.28 (-0.54, -0.02) |  |
| Area of current Residence | Urban | 3.75 (0.82) | - | 0.06 | 4.36 (0.76) | - | 0.90 | 4.23 (0.77) | - | 0.26 |
|  | Rural | 3.94 (0.65) | 0.19 (-0.005, 0.38) |  | 4.38 (0.67) | 0.01 (-0.19, 0.22) |  | 4.37 (0.55) | 0.15 (-0.11, 0.40) |  |
| COVID-19 self-reported symptoms at enrolment | No | 3.78 (0.80) | - | 0.85 | 4.36 (0.74) | - | 0.62 | 4.25 (0.75) | - | 0.75 |
|  | Yes | 3.74 (0.81) | -0.04 (-0.48, 0.40) |  | 4.48 (0.80) | 0.12 (-0.35, 0.59) |  | 4.35 (0.51) | 0.11 (-0.55, 0.77) |  |
| Body Mass Index at enrolment | Underweight | 3.81 (0.81) | 0.01 (-0.21, 0.23) | 0.24 | 4.3 (0.82) | -0.06 (-0.28, 0.17) | 0.61 | 4.21 (0.77) | -0.05 (-0.32, 0.23) | 0.91 |
|  | Normal | 3.80 (0.76) | - |  | 4.36 (0.76) | - |  | 4.26 (0.75) | - |  |
|  | Overweight and above | 3.59 (1.05) | -0.21 (-0.46, 0.04) |  | 4.46 (0.50) | 0.10 (-0.15, 0.35) |  | 4.20 (0.56) | -0.06 (-0.41, 0.30) |  |

^#^Generated from simple linear regression. *Test for trend p-value. All participants who had been COVID-19-vaccinated prior to baseline were excluded from the analysis. In addition, participants who received vaccination during the study between months 6 and 12 were excluded from analyses at month 12.

**Supplementary Table 3: Associations between malaria exposures and SARS-CoV-2 seropositivity restricted to anti-nucleocapsid-positive participants**

| **Main malaria exposures** | | **Month 0** | | **Month 6** | | **Month 12** | |
| --- | --- | --- | --- | --- | --- | --- | --- |
|  |  | **Adjusted OR (CI)** | **p-value** | **Adjusted OR (CI)** | **p-value** | **Adjusted OR (CI)** | **p-value** |
| Clinical malaria from birth to 10 years | | 1.40 (0.94, 2.08) | 0.10 | 1.46 (0.89, 2.39) | 0.13 | 0.82 (0.45, 1.51) | 0.53 |
| Asymptomatic malaria at ages 1-5 and 9 years | | 0.95 (0.53, 1.71) | 0.87 | 0.90 (0.45, 1.81) | 0.77 | 0.66 (0.29, 1.50) | 0.33 |
| Clinical malaria Episodes from birth to 10 years | None | REF | 0.36 | REF | 0.25 | REF | 0.76 |
|  | 1 episode | 1.31 (0.77, 2.22) |  | 1.73 (0.86, 3.48) |  | 0.67 (0.31, 1.46) |  |
|  | 2 – 4 episodes | 1.35 (0.83, 2.20) |  | 1.65 (0.86, 3.17) |  | 0.88 (0.43, 1.83) |  |
|  | >4 episodes | 1.63 (0.91, 2.92) |  | 1.04 (0.53, 2.05) |  | 1.03 (0.39, 2.74) |  |
| Malaria serology at age 13–17 years | PfMSP-2 | 1.45 (0.56, 3.73) | 0.44 | 1.84 (0.46, 7.35) | 0.38 | 1.86 (0.09, 40.90) | 0.69 |
|  | PfAMA-1 | 0.87 (0.62, 1.27) | 0.51 | 0.92 (0.57, 1.49) | 0.74 | 1.41 (0.57, 3.49) | 0.46 |

* All values generated by multivariable logistic regression and adjusted for age, sex, socioeconomic status, history of clinically diagnosed allergy, and BCG strain at birth. All participants who had been COVID-19-vaccinated prior to baseline were excluded from the analysis. In addition, participants who received vaccination during the study between months 6 and 12 were excluded from analyses at month 12.
